# Impact of Endometrial Preparation on the Maternal and Fetal Cardiovascular Variables of the First Trimester Combined Screening Test

**DOI:** 10.3390/jcm12216854

**Published:** 2023-10-30

**Authors:** Chiara Dallagiovanna, Laura Benaglia, Marco Reschini, Luca Di Gesaro, Letizia Li Piani, Nicola Persico, Paola Vigano’, Edgardo Somigliana

**Affiliations:** 1 Infertility Unit, Fondazione IRCCS Ca’ Granda Ospedale Maggiore Policlinico, 20122 Milan, Italy; chiara.dallagiovanna@policlinico.mi.it (C.D.); laura.benaglia@policlinico.mi.it (L.B.); ludiges@gmail.com (L.D.G.); letizia.lipiani@unimi.it (L.L.P.); paola.vigano@policlinico.mi.it (P.V.); edgardo.somigliana@policlinico.mi.it (E.S.); 2 Department of Clinical Sciences and Community Health, Università Degli Studi di Milano, 20122 Milan, Italy; nicola.persico@policlinico.mi.it; 3 Fetal Medicine and Surgery Service, Fondazione IRCCS Ca’ Granda Ospedale Maggiore Policlinico, 20122 Milan, Italy

**Keywords:** in vitro fertilization, frozen embryo, first trimester screening test, natural cycle, HRT

## Abstract

The modality of endometrial preparation for the transfer of frozen-thawed embryos may influence maternal and fetal adaptation to pregnancy and could thus impact the results of the first trimester combined screening test. We conducted a retrospective cross-sectional study on singleton pregnancies achieved by embryo transfer of a single frozen-thawed blastocyst, comparing two different endometrial preparation protocols: natural cycle (*n* = 174) and hormone replacement therapy (HRT) (*n* = 122). The primary outcome was the risk of preeclampsia at the first trimester combined screening test. Secondary endpoints included variable reflecting fetal cardiac function (nuchal translucency and fetal heart rate), maternal adaptation (median arterial blood pressure—MAP and uterine arteries pulsatility index—UtA-PI), and placentation (pregnancy associated plasma protein A and placental growth factor). The risk of early preeclampsia was comparable in the two groups (38% vs. a 28%, *p* = 0.12). However, women in the natural cycle group showed lower fetal heart rate (159 [155–164] vs. 164 [158–168], *p* = 0.002) and higher UtA-PI (0.96 [0.74–1.18] vs. 0.72 [0.58–0.90], *p* < 0.001). The frequency of a screening test at high risk for aneuploidies was similar. The modality of transfer of frozen-thawed embryos is associated with changes in the variables reflecting maternal and fetal cardiovascular function.

## 1. Introduction

In recent years, the frozen embryo transfer (FET) technique has become more and more widespread, accounting for the 35% of all the homologous in vitro fertilization (IVF) procedures performed in Europe in 2018 [1]. Improvements in freezing techniques played a role but, most importantly, indications for FET have expanded. This strategy allows to reduce the risk of multiple pregnancies by facilitating single embryo transfer, it increases the chance of pregnancy when early elevation of progesterone levels occurs during fresh cycles, it reduces the risk of ovarian hyperstimulation syndrome, and it allows to perform preimplantation genetic testing [2].

It is still a matter of debate which is the best endometrial preparation for patients undergoing FET [3,4]. Two main approaches are used, the ovulatory-based cycle and the use of hormone replacement therapy (HRT) [5]. The former can be obtained by the natural ovulatory cycle (detecting of spontaneous ovulation by ultrasound monitoring and urine tests for identifying LH surge), modified natural cycle (administration of human chorionic gonadotropins—hCG—once the dominant follicle has reached appropriate development), or mild ovarian stimulation (administering gonadotropins or aromatase inhibitor, and then hCG). In the HRT cycles, ovulation is avoided, and preparation is achieved by administering estrogens to obtain adequate thickening of the endometrium, and subsequently adding progesterone (typically at high doses) to promote endometrial receptivity. Progesterone is also frequently given for natural cycles transfers, even if at lower doses [6]. At present, HRT is the most frequently employed, since it allows scheduling the embryo transfer, thus becoming more convenient for both patients and doctors.

Previous studies that compared natural conceptions and FET pregnancies suggested that there are differences in components of first trimester combined screening test for fetal aneuploidies: differences emerged for nuchal translucency (NT) thickness, and maternal serum levels of free beta-human chorionic gonadotropin (free β-hCG) and pregnancy-associated plasma protein-A (PAPP-A) [7,8,9]. Moreover, preeclampsia and intrauterine growth restriction are more common in IVF pregnancies compared to natural conceptions [10,11,12], an effect attributable to an altered maternal and fetal cardiovascular adaptation [13].

Very recently, the scientific community has also begun to question the possible effects of the modality of endometrial preparation for transfer of frozen embryos on the course of pregnancy and on fetal health [14,15,16]. Transfer of frozen embryo on natural cycle could reduce the risk of pregnancy complications compared to HRT, including the incidence of hypertensive disorders of pregnancy and preeclampsia [14,16]. It has been hypothesized that a key role is played by the corpus luteum and its hormones, especially relaxin, a potent vasodilator involved in cardiovascular and renal adaptation in pregnancy, which is able to cross the placenta, thus influencing both the maternal and the fetal side [17,18].

In this scenario, it seems plausible that the modality of endometrial preparation may influence both maternal and fetal cardiovascular adaptation, with repercussions on the calculated risk of preeclampsia (and possibly the risk of aneuploidies) in the screening assessment performed early in pregnancy. The aim of the present study was to retrospectively compare first trimester ultrasound screening test components in women treated with natural cycles and in those receiving HRT, particularly focusing on fetal cardiovascular variables (fetal heart rate—FHR and NT), maternal adaptation variables (uterine artery pulsatility index—UtA-PI and blood pressure), and placentation (PAPP-A and placental growth factor—PIGF).

## 2. Materials and Methods

A single-center, retrospective, cross-sectional study was conducted at the Infertility Unit and the Fetal Medicine and Surgery Service of Fondazione IRCCS Ca’ Granda Ospedale Maggiore Policlinico in Milan, Italy. The study focused on pregnancies resulting from the transfer of frozen-thawed blastocysts that occurred between January 2017 and December 2019. Patient identification was carried out using the Meditex software (Critex GmbH, Regensburg, Germany) and the Astraia software (Astraia Software GmbH Occamstr, Munich, Germany). The inclusion criteria were defined as follows: (1) the transfer of a single frozen-thawed blastocyst, (2) the successful attainment of a singleton pregnancy progressing beyond the first trimester, and (3) the performance of the first trimester screening test at our center. Women who satisfied all eligibility criteria and experienced more than one pregnancy with frozen embryos during the study period were included only for their first pregnancy. Multiple pregnancies, therapeutic abortion (due to aneuploidies or fetal malformations), or fetal malformations detected later in pregnancy or at birth were excluded. Women performing IVF in other centers could be included provided that complete information on their IVF cycle was available. The women enrolled in the study were categorized into two groups based on their endometrial preparation protocols: (1) the natural cycle (all types) group, and (2) the programmed cycle with hormone replacement therapy (HRT) group. The study received approval from the local Ethical Committee (Comitato Etico Milano Area B, Protocol Number 1105_2019). Informed consent was not required due to the retrospective nature of the study. Nevertheless, it is important to note that all women seeking services at our facilities routinely provide informed consent for the use of their data in research.

Data on demographic and clinical characteristics, details of IVF procedures performed, and information on pregnancy outcome were extracted from patients’ clinical and biological charts, as well as from Meditex and Astraia softwares. Furthermore, in accordance with the Italian local regulations (20/2004), a proactive monitoring of all pregnancies in proximity to their delivery dates was consistently conducted. Information on the course of the pregnancy was obtained from medical records, for those patients who delivered in our hospital, or through phone calls using a standardized questionnaire, for those patients who delivered elsewhere.

The primary outcome was the frequency of a high-risk condition for preeclampsia at the first trimester screening test. Secondary endpoints were differences in variables indicative of fetal cardiac function (FHR and NT), maternal adaptation (arterial blood pressure and UtA-PI), and placentation (PAPP-A and PIGF).

For women undergoing IVF in our center, patients’ distribution between the two groups (natural cycle or HRT) was primarily made by assessing the individual’s medical history [19]. The natural cycle protocol was designed for women with regular menstrual cycles, typically lasting between 24 and 35 days. Hormone replacement therapy (HRT) was administered to those with irregular menstrual cycles or those who were unable to detect the LH surge in a previous cycle. Additionally, HRT was considered for women facing logistical challenges, such as those residing in distant areas. Comprehensive details of the endometrial preparation protocols can be found in a separate document [19]. Indications to HRT vs. natural cycle for women who received ART in other units differed, with some centers following our same policy and others scheduling all women to HRT. For those using natural cycle, however, the regimen differed, being done on pure natural cycle without adding hCG or progesterone in our unit [19] while being obtained with modified natural cycles (administration of hCG at subsequent progesterone supplementation) in all other centers.

The First trimester screening test was performed according to the Fetal Medicine Foundation Guidelines [20,21]. Maternal blood samples were collected between 11+3 and 12+6 gestational weeks to analyze free β-hCG, PAPP-A, and PIGF serum levels. Maternal serum biochemical markers were then converted into multiple of the median (MoM) using the Fetal Medicine Foundation algorithm 2012 (version 2.7) included in the Astraia software. Transabdominal or transvaginal ultrasound (when the transabdominal image was not adequate) was performed between 11 + 0 and 13 + 6 gestational weeks. All the operators who performed this investigation were certified by the Fetal Medicine Foundation (United Kingdom) and conducted the ultrasound evaluation according to the recommended criteria: a sagittal section of the fetus is needed; the image must be magnified for the fetus to occupy 75% of the screen; fetal skin must be distinguished from amnion; the maximum thickness of the nuchal translucency, which is the space between the fetal skin and the soft tissues covering the cervical spine, must be measured [22].

The screening for preeclampsia was performed combining maternal risk factors, measurements of mean arterial pressure (MAP), maternal serum PAPP-A, PIGF, and UtA-PI [23,24]. The risk for preeclampsia was considered high (or positive) when greater than 1 in 100, according to the Fetal Medicine Foundation guidelines [25]. The risk for fetal trisomies 21, 18, and 13 was then performed by combining maternal age, fetal NT thickness, FHR, and maternal serum free β-human chorionic gonadotropin (hCG) and PAPP-A [22]. The risk for trisomies 21, 18, 13 was considered high when greater than 1 in 250, according to the Essential Levels of Care (LEA) of the Italian Ministry of Health.

The collected data were analyzed using Statistical Package for Social Science (SPSS 23.0, IBM Corp, Armonk, NY, USA). Differences between the two groups were assessed using appropriate statistical tests, including the Fisher exact test, Chi-square test, Student *t*-test, or Mann–Whitney test. The normality of data distribution was evaluated using the Shapiro–Wilk test, and non-normally distributed variables were compared using non-parametric statistics. Continuous variables were presented as mean ± standard deviation (SD) or median [interquartile range (IQR)], while categorical variables were expressed as frequencies and percentages. Baseline variables that exhibited differences between the study groups were included in a multivariate logistic model to calculate the adjusted odds ratio (OR) for preeclampsia. Statistically significant results were defined as *p* values less than 0.05.

The scheduled sample size was at least 200 women. Knowing in advance that the ratio between natural cycle and HRT was about 1:1, the sample size was calculated stating as clinically relevant an increased risk of preeclampsia in the first trimester screening test of 25% in HRT group vs. 10% in natural cycle group. Type I and II errors were set at 0.05 and 0.20, respectively. The recruitment period was set between January 2017 and July 2019 to allow for this goal.

## 3. Results

**Figure 1:**
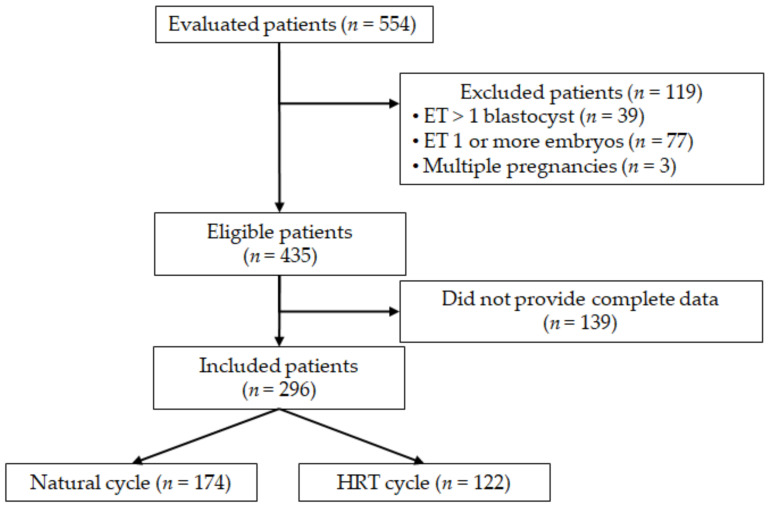
Flow-chart of the study.

A total number of 554 women who had performed FET cycles underwent the first trimester screening test at our hospital and were thus considered for inclusion in the study. Among these, 435 women met the inclusion criteria and were therefore contacted by telephone to obtain the necessary data regarding the IVF cycle. Complete data were not available in 139 cases, who were consequently excluded. Overall, 296 women were included: 174 women underwent natural cycle monitoring, while 122 women received HRT (Figure 1).

Table 1 provides a summary of the baseline characteristics for both groups. There were no significant differences in women age, BMI, smoking status, number of previous pregnancies, number of IVF cycles performed, and number of gynecological surgical procedures undergone. As expected, women in the natural cycle group had regular cycles more frequently than women in HRT group (93% vs. 74%, *p* < 0.001), this being the prerequisite for enrollment in the first group. The rate of egg donations also differed, as expected, since women belonging to the HRT group have a higher frequency of irregular menstrual cycles or frank amenorrhea (20% vs. 3%, *p* < 0.001).

Table 2 shows maternal and fetal variables included in the first trimester screening test for the evaluation of the risk of trisomies and preeclampsia. No differences emerged in maternal serum biochemical markers (β-hCG, PAPP-A, and PlGF), and NT thickness. Accordingly, no differences emerged in the frequency of high risk first trimester combined test for trisomies 21, 18, and 13.

The screen positive rate for preterm preeclampsia was comparable in the two groups (Table 2), being 38% and 28% in women treated with natural cycle and HRT, respectively (*p* = 0.12). The crude OR of being at risk for preeclampsia in women treated with the natural cycle was 1.57 (95%CI: 0.93–2.65]. The OR adjusted for age, parity, oocytes donation, and anovulation was 1.54 (95%CI: 0.88–2.71).

**Figure 2:**
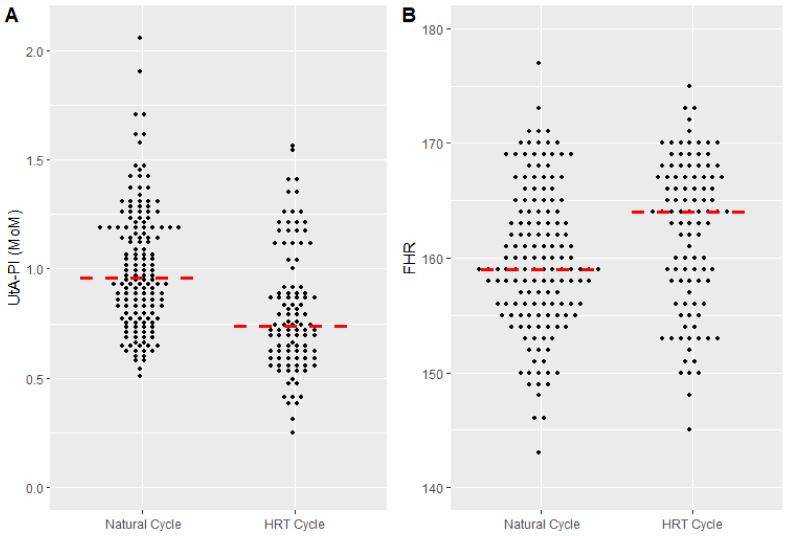
(**A**) Uterine artery pulsatility index (UtA-PI) in women treated with natural cycle and hormone replacement therapy (HRT). Data are reported in multiples of the median (MoM). The median [interquartile range] is 0.94 [0.74–1.18] vs. 0.72 [0.58–0.90] (*p* < 0.001). (**B**) Fetal heart rate (FHR) in women treated with natural cycle and hormone replacement therapy (HRT). The median [interquartile range] is 159 [155–164] and 164 [158–168], respectively (*p* = 0.002).

However, some variables significantly differed (Table 2). Women in the HRT cycle group had lower UtA-PI MoMs, when compared to women in natural group (0.72 [0.60–0.92] vs. 0.94 [0.78–1.19], *p* < 0.001), which showed values in the normal range (Figure 2A). FHR was significantly higher in the HRT group compared to natural cycle group (164 [158–167] vs. 159 [156–165], *p* = 0.002) (Figure 2B).

All the analyses were repeated including only homologous IVF (excluding the 25 pregnancies obtained with egg donation). Results were very similar (Supplementary Tables S1 and S2). Finally, the analyses were repeated including only women treated in our center (157). Again, no substantial differences emerged. 

Table 3 shows pregnancy and neonatal outcomes. No differences emerged in the rate of pregnancy and neonatal complications between the two groups. Looking specifically at hypertensive disorders in pregnancy, no difference emerged between the two groups. The incidence of preeclampsia was overlapping in the two groups (2% in the natural group vs. 6% in HRT group, *p* = 0.10) and comparable to the general population (2–8%) (Table 3).

## 4. Discussion

This retrospective study, conducted on 296 cycles of frozen-thawed blastocysts, aimed to evaluate the effect of endometrial preparation protocols on the calculated risk of preeclampsia in the first trimester combined screening test. We failed to highlight a different risk, but women undergoing HRT showed a lower UtA-PI, and higher FHR, when compared to women in the natural group. Yet, the risk of preeclampsia was equivalent in the two groups, probably because these statistically significant variables act in opposite ways on the risk of preeclampsia. Conversely, no differences emerged in all the components of the risk calculation algorithm for aneuploidies: maternal serum levels of β-hCG and PAPP-A, and NT thickness did not differ. The overall risk of aneuploidy was unaffected. Finally, no differences emerged in the incidence of obstetric and neonatal complications, with the incidence of preeclampsia being overlapping in the two groups and comparable to the incidence in the general population.

Our study generally confirmed the tested hypothesis, i.e., that the modality of endometrial preparation for FET could influence fetal and maternal cardiovascular variables already at first trimester screening. The observation that women treated with HRT had a lower UtA-PI (thus reduced resistance compared to natural cycle) was, however, quite unexpected and paradoxical considering what has been observed so far in the literature [16]. An increased resistance of uterine arteries is thought to predispose to preeclampsia development latter in pregnancy. Corpus luteum plays a crucial function in the early stages of pregnancy, by producing not only progesterone but also a plethora of hormones necessary for implantation, placentation, and maintenance of pregnancy. Among these, relaxin is a protein hormone with a vasodilating action that is involved in hemodynamic changes that occur in early pregnancy, contributing to cardiovascular and renal adaptation [14,26]. Our finding on UtA-PI seems therefore to suggest a protective effect of HRT with respect to the risk of preeclampsia.

Reasons to explain inconsistencies of our findings with previous literature and with notions of pregnancy physiology were not actively investigated in our study. However, some speculations can be put forward. First, one could hypothesize that differences in the baseline characteristics of the subjects included in the available studies could play a role. Notably, in a previous study of our group aimed at investigating the incidence of hypertensive disorders (rather than the results of the screening test) in women receiving HRT or natural cycle, we failed to show any difference [15]. Our local population may differ in terms of risk factors for preeclampsia compared to other Western populations. Obesity, for instance, plays an important adjuvant role in the determinism of preeclampsia and was uncommon in our population (Table 1). Overall, one may conclude that the protective effect of the corpus luteum may be clinically relevant only in women with additional risk factors for preeclampsia. Second, the study is not randomized and selection biases in the allocation of HRT versus natural cycle could play a role. HRT was more commonly used in women with irregular cycles (typically affected by polycystic ovarian syndrome) or requiring oocytes donation. Both conditions may be per se associated with vascular changes and increased risk of hypertensive disorders. However, this cannot explain our paradoxical findings. Moreover, excluding women receiving egg donation did not change the results. Third, and possibly most intriguing, we wondered whether the paradoxical lower resistance in women treated with HRT could be consequent to the administration of vaginal progesterone at high doses. It has been widely demonstrated that progesterone exerts a vasodilatory effect through the relaxation of smooth muscle fibers, which results in a decrease in uterine arteries impedance [27]. The exact mechanism of action of progesterone is currently unknown. Genomic and non-genomic mechanisms have been hypothesized [28]. This effect is especially exacerbated by vaginal progesterone, since the vaginal route guarantees higher uterine bioavailability, allowing a rapid absorption and avoiding first pass hepatic effect [29]. Of relevance here is that, in the HRT cycle, progesterone is typically administered vaginally and at high dose (typically 800 mg per day). In the natural cycle, it is not given or, when given, the doses are lower [6]. Overall, if valid, this interpretation would also question the direct effect of the increased resistance of the uterine vessels in the development of preeclampsia later in pregnancy. In fact, this phenomenon may be an indirect epiphenomenon of a disrupted process of implantation rather than being a causative effect. One may speculate that vaginal progesterone may mask these early predictors of preeclampsia. It cannot, however, influence the effect of the presence of the corpus luteum on systemic blood pressure (that conversely was higher in the HRT group). These two effects go in opposite directions, and this can explain why the overall risk of preeclampsia in the first trimester screening test is not different between the two groups. Albeit intriguing, our hypothesis is speculative, and further studies are required to clarify this issue.

Analyzing variables of fetal cardiac function, a difference in FHR emerged, which was higher in the HRT group. This difference might suggest a different cardiac function in the fetuses of patients undergoing HRT, as suggested by Valenzuela-Alcaraz et al., who hypothesized a different cardiac remodeling in IVF pregnancies [30]. However, to date, it has not been clarified what aspects of the IVF technique may be involved in these modifications. Our study suggests that endometrial preparation could be one of these factors. Hormones produced by corpus luteum such as relaxin may somehow also influence the development of the fetal cardiovascular apparatus, the impact on FHR being only one biomarker of this effect.

No differences emerged in our study from the analysis of the different components of the first trimester combined screening test for aneuploidies between the two study groups. Previous published studies showed alterations in maternal serum markers of aneuploidies, though it has not been clarified yet whether this association could be attributed to the underlying infertility or the IVF procedures themselves [9]. In particular, the effect of IVF techniques on maternal serum PAPP-A levels is controversial. Our study does not provide new information on the possible effects of IVF in general, it just suggests that the modality of endometrial preparation should not be considered an additional confounder.

Unaffected IVF pregnancies were shown to be associated with lower levels of maternal serum PAPP-A levels, when compared to natural pregnancies [7]. Comparing fresh and frozen-thawed embryo transfer, Amor et al. observed lower maternal serum PAPP-A in pregnancies achieved by fresh embryo transfer [31]. Focusing on the effect of hormonal treatment on PAPP-A maternal serum level, the same group showed that hormonal therapies were associated with lower PAPP-A levels [31]. The authors hypothesized that exogenous hormones administered during IVF treatments may interfere with the endocrine changes occurring early in pregnancy, thus causing lower PAPP-A levels [31]. Lower PAPP-A levels are associated with a higher risk on aneuploidies in the first trimester combined screening test and consequently with a higher rate of chorious villus sampling and amniocentesis. However, PAPP-A levels represent an alarm bell not only for trisomies, but for many other obstetrical complications (i.e., gestational hypertension, preeclampsia, gestational diabetes) and perinatal adverse outcomes (prematurity, low birth weight, neonatal morbidity, and mortality) [32,33,34].

Regarding β-hCG levels, our study did not highlight differences between the two study groups, similarly to what was previously observed in the literature [7,35], and analysis of NT thickness did not reveal differences between the two groups. This aspect has not yet been fully clarified in the literature: although most studies do not document differences in NT thickness between natural and IVF pregnancies [36,37,38,39], there are still studies that have shown an increase in NT thickness [40] and studies that have shown its reduction in IVF pregnancies [7], compared to natural conceptions.

Some limitations of our study deserve to be mentioned. This is a retrospective study, and thus was exposed to all the inaccuracies of this study design. Although large, the study was not powered to detect milder but potentially clinically interesting differences. We thus focused solely on the risk of pregnancy calculated in the first trimester screening test, and not on preeclampsia per se or on other obstetric outcomes. Much larger studies are required to address this issue. Findings emerging from secondary analyses (such as those on the risk of aneuploidy) should be viewed as exploratory, since not included in the primary outcome (increased risk of type I error). Moreover, this study includes subjects treated in different infertility centers that may differ in the mode of allocation and protocols used. This inevitably makes our cohort less uniform and more exposed to confounders. Finally, given that allocation was mainly guided by clinical variables (regular cycles), the two populations may differ for several and complex baseline characteristics that were not captured in our study.

## 5. Conclusions

The present study did not show differences in the first trimester combined screening test for preeclampsia or aneuploidy between women undergoing different endometrial preparation protocols. However, differences emerged from the analyses of some variables reflecting maternal and fetal cardiovascular function. The most striking and surprising observation is the lower resistance of the uterine arteries in HRT cycles, a finding that contrasts with the theory of the protective role of the corpus luteum on the risk of preeclampsia. This is possibly due to an effect of vaginal progesterone on uterine arteries blood flow, a kind of artefact consequent to the vaginal administration of very high doses of the hormone. However, this effect may not temper the detrimental effects of HRT on the risk of preeclampsia. Further evidence is needed to better understand this intriguing finding and clarify its clinical relevance.

## Figures and Tables

**Table 1:** Baseline characteristics of the two study groups.

Characteristics	Natural Cycle	HRT Cycle	*p*
	*n* = 174	*n* = 122	
Age (years)	36 [33–39]	37 [33–41]	0.08
BMI (Kg/m^2^)	21.3 [19.6–23.4]	20.7 [19.4–23.1]	0.28
Smoking	28 (16%)	15 (12%)	0.41
Previous deliveries	35 (20%)	19 (16%)	0.36
Previous pregnancy	65 (37%)	52 (43%)	0.40
Duration of infertility (years)	3 [2–4]	3 [2–4]	0.33
Previous gynecology surgery	42 (24%)	30 (25%)	1.00
Type of IVF			<0.001
Homologous	168 (97%)	97 (80%)	
Heterologous	6 (3%)	25 (20%)	
Regular cycles	162 (93%)	90 (74%)	<0.001
Indication to IVF			0.004
Unexplained	56 (32%)	44 (36%)	
Endometriosis	20 (12%)	7 (6%)	
Tubal factor	16 (9%)	6 (5%)	
Disovulatory	4 (2%)	16 (13%)	
Male factor	53 (31%)	29 (24%)	
Genetic	13 (7%)	13 (10%)	
Mixed	12 (7%)	7 (6%)	

Data are presented as median (interquartile range—IQR) or number (%).

**Table 2:** Maternal and fetal parameters in first trimester combined screening test.

Characteristics	Natural Cycle	HRT Cycle	*p*
	*n* = 174	*n* = 122	
β-hCG (MoM)	1.14 [0.76–1.58]	1.11 [0.75–1.77]	0.56
PAPP_A (MoM)	1.21 [0.71–1.73]	1.19 [0.82–1.92]	0.23
NT	1.8 [1.6–2.0]	1.8 [1.5–2.0]	0.45
FHR	159 [155–164]	164 [158–168]	0.002
UtA-PI (MoM)	0.94 [0.74–1.18]	0.72 [0.58–0.90]	<0.001
MAP (MoM)	1.021 [0.964–1.087]	1.041 [0.980–1.111]	0.039
PIGF (MoM)	1.04 [0.80–1.19]	1.03 [0.72–1.37]	0.83
High risk for trisomy 21 (≤1 su 250)	13 (8%)	11 (9%)	0.67
High risk for trisomy 18 (≤1 su 250)	2 (1%)	2 (2%)	1.00
High risk for trisomy 13 (≤1 su 250)	1 (1%)	2 (2%)	0.57
High risk combined test	15 (9%)	12 (10%)	0.84
High risk for preeclampsia ^a^	61 (38%)	31 (28%)	0.12

Data are presented as median (interquartile range—IQR) or number (%). ^a^ Data available for 159 patients in natural cycle group and 109 in HRT group.

**Table 3:** Pregnancy and neonatal outcomes.

Characteristics	Natural Cycle	HRT Cycle	*p*
	*n* = 174	*n* = 122	
Therapeutic abortion ^a^	5 (3%)	2 (2%)	0.70
**Live birth**	169	120	
Preterm deliveries (<37 weeks)	13 (8%)	11 (9%)	0.67
Neonatal weight <2500 g	10 (6%)	9 (8%)	0.64
Hypertensive disorders in pregnancy	8 (5%)	12 (10%)	0.10
Preeclampsia	3 (2%)	7 (6%)	0.10
Gestational diabetes	5 (3%)	5 (4%)	0.75
Other obstetrical complications ^b^	8 (5%)	8 (7%)	0.60
Neonatal complications ^c^	4 (2%)	6 (5%)	0.33

Data are presented as number (%). ^a^ Pregnancy terminations were decided because of multiple malformations and chromosome 21 aneuploidy (natural cycle) and complete agenesis of the Corpus callosum, chromosome aneuploidy, and fetal heart disease (HRT group). ^b^ Obstetrical complications include 3 women with placenta previa, 2 cholestasis, 1 hemorrhage, 1 cervico-segmental incontinence, and 1 placental abruption in the natural cycle group. In the HRT group, obstetrical complications include 3 pregnancies complicated cholestasis, 2 premature ruptures of fetal membranes, 2 polyhydramnios, and 1 placenta previa. ^c^ Neonatal complications include ileal atresia, cerebral hemorrhage, horseshoe kidney, and 1 infant died of cardiac abnormalities in the natural cycle group. In the HRT group, neonatal complications include bronchodysplasia, ichthyosis vulgaris, neurofibromatosis, Kabuki syndrome, cleft palate, and club foot.

## Data Availability

Data available upon request.

## Supplementary Materials

The following supporting information can be downloaded at: https://www.mdpi.com/article/10.3390/jcm12216854/s1, Table S1: Baseline characteristics of the two study groups (only homologous); Table S2: Maternal and fetal parameters in first trimester combined screening test (only homologous).
