# Supplementary material for: Impact of Endometrial Preparation on the Maternal and Fetal Cardiovascular Variables of the First Trimester Combined Screening Test
Source: J Clin Med. 2023 Oct 30;12(21):6854. doi: 10.3390/jcm12216854 (PMC10650935; doi:10.3390/jcm12216854)
Supplement: Supplementary file 1 [file jcm-12-06854-s001.zip › jcm-2638755-supplementary.pdf]

Table S1. Baseline characteristics of the two study groups (only homologous).

Characteristics	Natural Cycle n = 168	HRT Cycle n = 97	p
Age (years)	36 [33—39]	36 [33–39]	0.84
BMI (kg/m^2)	21.3 [19.6–23.4]	20.8 [19.4–23.5]	0.46
Smoking	28 (17%)	13 (13%)	0.60
Previous deliveries	34 (20%)	14 (14%)	0.25
Previous pregnancy	63 (38%)	36 (37%)	1.00
Duration of infertility (years)	3 [2–4]	3 [2–4]	0.37
Previous gynecology surgery	40 (24%)	23 (24%)	1.00
Regular cycles	158 (94%)	69 (71%)	< 0.001
Indication to IVF			0.008
Unexplained	53 (32%)	31 (32%)	
Endometriosis	20 (12%)	5 (5%)	
Tubal factor	16 (9%)	6 (6%)	
Disovulatory	4 (2%)	14 (15%)	
Male factor	52 (31%)	28 (29%)	
Genetic	13 (8%)	6 (6%)	
Mixed	10 (6%)	7 (7%)	

Data are presented as median (interquartile range—IQR) or number (%).

Table S2. Maternal and fetal parameters in first trimester combined screening test (only homologous)

Characteristics	Natural Cycle n = 168	HRT Cycle n = 97	p
β-hCG (MoM)	1.13 [0.73–1.55]	1.09 [0.75–1.74]	0.76
PAPP_A (MoM)	1.21 [0.69–1.71]	1.17 [0.79–1.93]	0.35
NT	1.8 [1.6–2.0]	1.8 [1.6–2.0]	0.78
FHR	160 [155–164]	164 [157–167]	0.008
UtA-PI (MoM)	0.94 [0.76–1.19]	0.74 [0.58 – 0.94]	< 0.001
MAP (MoM)	1.019 [0.964–1.082]	1.040 [0.980–1.109]	0.035
PIGF (MoM)	1.04 [0.80–1.19]	1.03 [0.69–1.37]	0.80
High risk for trisomy 21 (≤ 1 su 250)	13 (8%)	10 (10%)	0.50
High risk for trisomy 18 (≤ 1 su 250)	2 (1%)	2 (2%)	0.63
High risk for trisomy 13 (≤ 1 su 250)	1 (1%)	2 (2%)	0.56
High risk combined test	15 (9%)	11 (11%)	0.53
High risk for preeclampsia[a]	59 (38%)	26 (29%)	0.17

Data are presented as median (interquartile range—IQR) or number (%).

[a] Data available for 154 patients in natural cycle group and 89 in HRT group.
